# Supplementary material for: Curcumin enhances bedaquiline’s efficacy against Mycobacterium abscessus: in vitro and in vivo evidence
Source: Microbiol Spectr. 2025 Mar 18;13(5):e02295-24. doi: 10.1128/spectrum.02295-24 (PMC12054065; doi:10.1128/spectrum.02295-24)
Supplement: Supplemental material — Fig. S1 to S6; Table S1. [file spectrum.02295-24-s0001.docx]

**Supplementary Materials:**


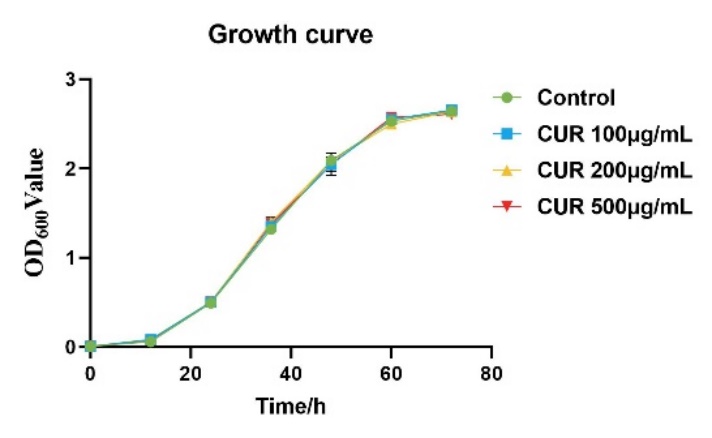


**Fig. S1. Growth curve of *M. abscessus* treated with different concentrations of curcumin (CUR) (n = 3).**


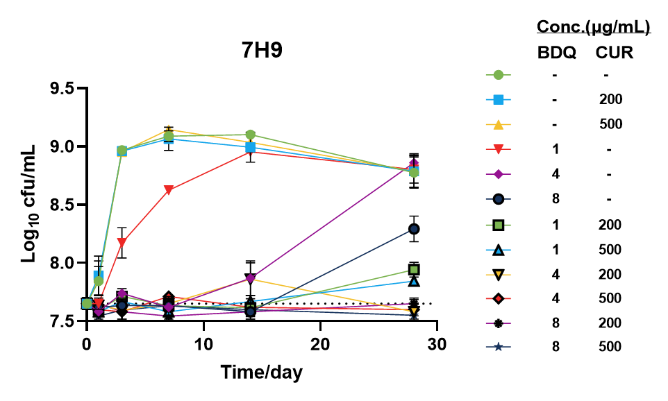


**Fig. S2.** **Time-kill kinetics of BDQ and CUR combinations against *M. abscessus* over 28 days.**

Killing kinetics of *M. abscessus* treated with bedaquiline (BDQ; 1, 4, and 8 μg/mL) and curcumin (CUR; 200 and 500 μg/mL), individually or in combination. Growth was quantified by CFU compared to a DMSO-treated control and monitored over 28 days. Data are shown as mean ± SD from triplicate cultures.


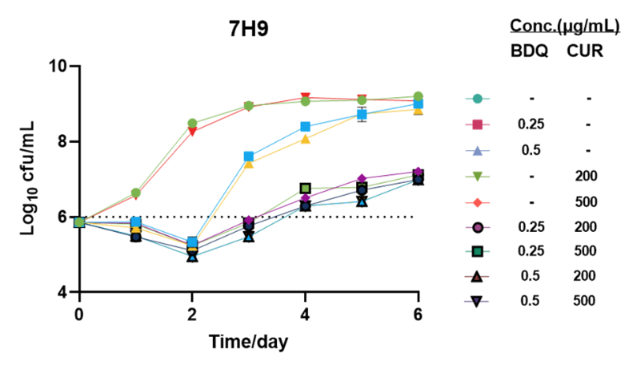


**Fig. S3. Time-kill kinetics of BDQ and CUR combinations against *M. abscessus* 6 days.**

Killing kinetics of *M. abscessus* treated with bedaquiline (BDQ; 0.25 and 0.5 μg/mL) and curcumin (CUR; 200 and 500 μg/mL), individually or in combination. Growth was quantified by CFU compared to a 20% DMSO-treated control and monitored over 6 days. Data are shown as mean ± SD from triplicate cultures.


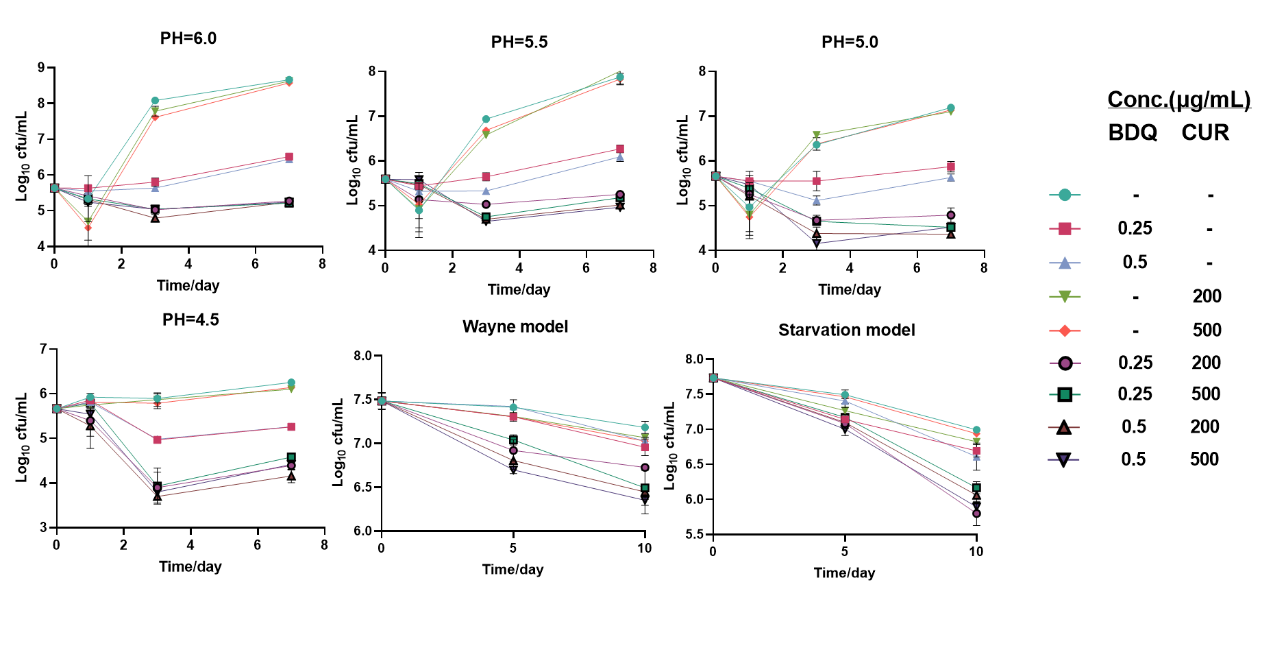


**Fig. S4. Survival curves of *M. abscessus* under acidic, hypoxic, and starvation conditions.**

Survival of *M. abscessus* under acidic stress (pH 6.0, 5.5, 5.0, and 4.5), hypoxic conditions (Wayne model), and nutrient-limited conditions (starvation model), treated with bedaquiline (BDQ; 0.25 μg/mL and 0.5 μg/mL) and curcumin (CUR; 200 μg/mL and 500 μg/mL), individually or in combination. An equivalent volume of 20% DMSO was used as a control. Survival was monitored for 7 days under acidic stress and for 10 days under hypoxic and starvation conditions.

**Fig. S5. Effect of BDQ and CUR combined treatment on *M. abscessus* infection in RAW264.7 macrophages.**

Viability of *M. abscessus* in RAW264.7 macrophages treated with bedaquiline (BDQ; 0.5 μg/mL and 1 μg/mL), curcumin (CUR; 200 μg/mL and 500 μg/mL), or their combination. An equivalent volume of 20% DMSO was used as a control. CFU counts were measured at 1 and 3 days post-treatment.

**Fig. S6. Cytotoxicity evaluation of CUR on RAW264.7 macrophages.**

The cytotoxicity of curcumin (CUR) on RAW264.7 macrophages was assessed using the Cell Counting Kit-8 (CCK-8) assay. Macrophages were treated with increasing concentrations of CUR for 24 hours, and cell viability was measured as a percentage of the untreated control.

**Table S1. Differential Metabolites of *M. abscessus* Treated with BDQ, CUR, and BDQ-CUR Combination.**

| **Strain** | **Compound Name** | **FC(Treatment/Control)** | **Log_2_FC** | **Regulation** | **P val** |
| --- | --- | --- | --- | --- | --- |
| BDQ vs NC | 2-Aminoacrylate | 0.45094271 | -1.148983937 | down | 0.013087859 |
|  | 3-Methyl-2-oxobutanoic acid | 2.458652758 | 1.297867993 | up | 0.009536702 |
|  | 3-Oxopropanoate | 0.078011432 | -3.680170627 | down | 0.022902095 |
|  | 5-Methylthioadenosine | 0.154697577 | -2.692477492 | down | 0.007993626 |
|  | Acetoacetyl-CoA | 0.528075372 | -0.921184235 | down | 0.007564658 |
|  | Acetylaminobutanal | 0.301160831 | -1.731393951 | down | 0.048833157 |
|  | Acetylornithine | 5.404747952 | 2.43422734 | up | 3.52429E-06 |
|  | Aconitate | 5.624220248 | 2.491653092 | up | 6.98277E-07 |
|  | Adenine | 0.096540861 | -3.372716499 | down | 0.00153106 |
|  | Adenosine | 0.211525942 | -2.241093485 | down | 0.025096026 |
|  | ADP | 0.397086169 | -1.332475983 | down | 0.021836111 |
|  | Alanine | 0.51448117 | -0.958809818 | down | 0.04639341 |
|  | Aminocyclopropane carboxylate | 0.525229059 | -0.928981358 | down | 0.002302653 |
|  | AMP | 0.054272948 | -4.203622903 | down | 0.005382818 |
|  | Arginine | 0.425979423 | -1.231144353 | down | 0.009990471 |
|  | Aspartate | 0.500601858 | -0.99826445 | down | 0.022846402 |
|  | beta-D-Glucose6-phosphate | 0.046478768 | -4.42728436 | down | 0.002806271 |
|  | Carbamoyl-L-aspartate | 0.336368422 | -1.571885821 | down | 0.018557103 |
|  | CDP | 0.25030648 | -1.998232452 | down | 0.002353749 |
|  | cGMP | 0.112002796 | -3.158393342 | down | 0.028371171 |
|  | CIR | 4.258456164 | 2.090330499 | up | 0.02025193 |
|  | cis-2-Methylaconitate | 0.160167667 | -2.64234515 | down | 0.014204326 |
|  | Cytosine | 0.466616961 | -1.099689348 | down | 0.005865805 |
|  | d-Ala-d-Ala | 0.361917435 | -1.466267486 | down | 0.003994904 |
|  | dAMP | 0.082672957 | -3.596440697 | down | 0.008022553 |
|  | Deoxyadenosine | 0.126949857 | -2.97766932 | down | 0.003592215 |
|  | Deoxyguanosine | 0.211525942 | -2.241093485 | down | 0.025096026 |
|  | Deoxyinosine | 0.131060384 | -2.931696429 | down | 0.017156222 |
|  | D-Erythrose 4-phosphate | 0.047158863 | -4.406327239 | down | 0.00264211 |
|  | dGMP | 0.054272948 | -4.203622903 | down | 0.005382818 |
|  | dTMP | 0.077303916 | -3.693314691 | down | 0.001517796 |
|  | dUDP | 0.085232777 | -3.552447858 | down | 0.009834134 |
|  | dUMP | 3.039574005 | 1.603869145 | up | 0.036245121 |
|  | F6P | 0.045210013 | -4.467213873 | down | 0.028360454 |
|  | FAD | 0.085666536 | -3.545124433 | down | 0.002886256 |
|  | FGAR | 0.197221664 | -2.342110058 | down | 0.011634081 |
|  | FMN | 0.070213758 | -3.832102449 | down | 0.001109344 |
|  | G1P | 0.045210013 | -4.467213873 | down | 0.028360454 |
|  | G6P | 0.046478768 | -4.42728436 | down | 0.002806271 |
|  | Galactitol | 0.111168748 | -3.169176825 | down | 0.004592801 |
|  | Galactonate | 0.140970291 | -2.826536939 | down | 0.023622306 |
|  | Galactono-1,4-lactone | 1.549414124 | 0.631722796 | up | 0.005887127 |
|  | gamma-L-Glutamyl-L-cysteine | 0.121802553 | -3.03738372 | down | 0.025216225 |
|  | GlcNAc-P | 0.052315596 | -4.256615092 | down | 0.005655905 |
|  | Glutamine | 0.293988004 | -1.766170809 | down | 0.01436193 |
|  | Glycine | 0.296899632 | -1.751952792 | down | 0.001363731 |
|  | GMP | 0.121561178 | -3.040245537 | down | 0.002713392 |
|  | Guanine | 0.144763842 | -2.788226788 | down | 0.001373296 |
|  | Homoserine | 0.260788167 | -1.939049684 | down | 0.000315733 |
|  | Hypoxanthine | 0.034146485 | -4.872119118 | down | 0.004236272 |
|  | IMP | 0.008125824 | -6.94327022 | down | 0.002366526 |
|  | Inosine | 0.034791964 | -4.845102088 | down | 0.003481063 |
|  | Lactaldehyde | 0.160883437 | -2.635912287 | down | 0.000860055 |
|  | Mannosylglycerate | 0.034791964 | -4.845102088 | down | 0.003481063 |
|  | Methionine | 0.049307659 | -4.34204442 | down | 0.001273424 |
|  | Methylcitrate | 6.403611852 | 2.678885863 | up | 0.001063128 |
|  | Methylmaleate | 5.563315323 | 2.475944878 | up | 1.95211E-06 |
|  | NAD+ | 0.122118062 | -3.033651494 | down | 0.01662409 |
|  | Nicotinamide | 5.184577932 | 2.374226546 | up | 0.009669098 |
|  | Nicotinate | 0.280963893 | -1.831543357 | down | 0.020328866 |
|  | Ornithine | 0.330359988 | -1.597889134 | down | 0.007129863 |
|  | OXG | 0.122577647 | -3.028232182 | down | 0.013331189 |
|  | pantothenate | 0.161973402 | -2.626171168 | down | 0.00127109 |
|  | Phe | 0.368026765 | -1.442117404 | down | 0.018518438 |
|  | Proline | 0.467878349 | -1.095794625 | down | 0.039534751 |
|  | Propenoyl-CoA | 0.443577981 | -1.172740344 | down | 0.038703634 |
|  | PYR | 0.083954659 | -3.574245799 | down | 0.044775357 |
|  | Riboflavin | 0.206786638 | -2.273785128 | down | 0.004440409 |
|  | S-Acetyldihydrolipoamide-E | 0.333497786 | -1.584250911 | down | 0.022859917 |
|  | Sedoheptulose 7-phosphate | 0.083778489 | -3.577276319 | down | 0.002933773 |
|  | SUC | 0.169393013 | -2.561553726 | down | 0.000763668 |
|  | Threonine | 0.260788167 | -1.939049684 | down | 0.000315733 |
|  | Thymidine | 0.140690728 | -2.829400839 | down | 0.000937283 |
|  | Thymine | 0.247927079 | -2.012012242 | down | 0.002150688 |
|  | Trehalose | 0.009957157 | -6.650050413 | down | 0.003445354 |
|  | Trp | 0.482023409 | -1.052824883 | down | 0.035986272 |
|  | Tyr | 0.183503119 | -2.446123513 | down | 0.011524909 |
|  | Ubiquinone | 0.141651398 | -2.819583259 | down | 0.016401338 |
|  | UDP | 0.045667779 | -4.452679555 | down | 0.011042518 |
|  | UDP-D-galactose | 0.045788934 | -4.448857198 | down | 0.010449803 |
|  | UDP-GlcNAc | 0.035276703 | -4.825140446 | down | 0.006625664 |
|  | UDP-glucose | 0.045788934 | -4.448857198 | down | 0.010449803 |
|  | UMP | 0.04045724 | -4.627458293 | down | 0.008306064 |
|  | Uracil | 0.214725417 | -2.219435122 | down | 0.000740487 |
|  | Urate | 0.4133886 | -1.274429492 | down | 0.00093662 |
|  | Uridine | 0.144603058 | -2.789830029 | down | 0.0045245 |
|  | Xanthine | 0.244719966 | -2.030796285 | down | 0.003447993 |
|  | Xanthosine | 0.232586584 | -2.104160214 | down | 0.000153457 |
| CUR vs NC | 2-Deoxy-D-ribose 1-phosphate | 3.471260411 | 1.795459599 | up | 0.00106119 |
|  | 2-Oxobutanoate | 0.387063972 | -1.369356067 | down | 0.03105515 |
|  | 6-Phospho-D-gluconate | 1.941865999 | 0.957443649 | up | 0.02267656 |
|  | Aconitate | 1.842053452 | 0.881314926 | up | 0.01812889 |
|  | Adenine | 0.516103296 | -0.954268251 | down | 0.00476549 |
|  | Aspartate | 0.624328819 | -0.679622033 | down | 0.04338042 |
|  | Deoxyinosine | 3.051333848 | 1.609440035 | up | 0.02490208 |
|  | Deoxyribose phosphate | 3.471260411 | 1.795459599 | up | 0.00106119 |
|  | FAD | 0.552871337 | -0.854984316 | down | 0.01461889 |
|  | Galactitol | 0.593208602 | -0.753388578 | down | 0.03413663 |
|  | Guanine | 0.500282549 | -0.999184966 | down | 0.00485322 |
|  | Hypoxanthine | 0.260436308 | -1.940997501 | down | 0.00410201 |
|  | IMP | 0.463299651 | -1.109982499 | down | 0.01000452 |
|  | Methionine | 0.465076755 | -1.104459259 | down | 0.0052422 |
|  | Methylmaleate | 1.846392604 | 0.88470935 | up | 0.01257644 |
|  | Nicotinate | 0.612624011 | -0.706926182 | down | 0.03683062 |
|  | Proline | 0.48772601 | -1.035857183 | down | 0.04686977 |
|  | Succinate semialdehyde | 0.387063972 | -1.369356067 | down | 0.03105515 |
|  | Thymine | 1.542583271 | 0.625348371 | up | 0.01734759 |
|  | UDP | 0.474547694 | -1.075375005 | down | 0.04555175 |
|  | UDP-GlcNAc | 0.468337905 | -1.094378287 | down | 0.02904607 |
|  | UMP | 0.499356309 | -1.001858495 | down | 0.04474575 |
|  | Uracil | 0.602931935 | -0.729932949 | down | 0.01479126 |
|  | Xanthine | 0.283559853 | -1.81827481 | down | 0.00251339 |
|  | Xanthosine | 0.593403851 | -0.752913805 | down | 0.00151102 |
|  | cis-2-Methylaconitate | 0.46667585 | -1.099507285 | down | 0.04616838 |
|  | dAMP | 0.506141411 | -0.98238758 | down | 0.03701237 |
|  | dGMP | 0.495834334 | -1.01206992 | down | 0.02666592 |
|  | dTMP | 0.481821404 | -1.053429612 | down | 0.00356022 |
|  | pantothenate | 0.411558873 | -1.280829274 | down | 0.00201136 |
|  | 2-Aminoacrylate | 0.562058681 | -0.831207335 | down | 0.02704083 |
|  | Acetoacetyl-CoA | 0.5197926 | -0.943992001 | down | 0.00676194 |
|  | Acetylaminobutanal | 0.630418833 | -0.66561746 | down | 0.04110663 |
|  | Alanine | 0.488269509 | -1.034250407 | down | 0.03850448 |
|  | Anthranilate | 0.512041902 | -0.965666218 | down | 0.03248556 |
|  | Cytosine | 0.627222595 | -0.672950562 | down | 0.02579371 |
|  | Glycine | 0.486293792 | -1.040099919 | down | 0.04417913 |
|  | Histidine | 0.566958656 | -0.818684561 | down | 0.03874423 |
|  | Homoserine | 0.605151247 | -0.72463233 | down | 0.02195689 |
|  | Lysine | 0.628031747 | -0.671090606 | down | 0.03470587 |
|  | Propanoyl-CoA | 1.882104931 | 0.912347064 | up | 0.00479793 |
|  | Threonine | 0.605151247 | -0.72463233 | down | 0.02195689 |
|  | Uridine | 37.75637591 | 5.238648388 | up | 0.00934946 |
|  | beta-D-Glucose6-phosphate | 0.611913377 | -0.708600657 | down | 0.04158651 |
|  | AMP | 0.495834334 | -1.01206992 | down | 0.02666592 |
|  | OXG | 0.384969152 | -1.377185249 | down | 0.03307759 |
| BC vs NC | 2-Aminoacrylate | 0.177485427 | -2.494227525 | down | 0.006245228 |
|  | 2-Deoxy-D-ribose 1-phosphate | 3.148765102 | 1.654786136 | up | 0.006540596 |
|  | 3-Oxopropanoate | 0.085487214 | -3.548147532 | down | 0.023418111 |
|  | 5-Methylthioadenosine | 0.024289618 | -5.363516407 | down | 0.005744566 |
|  | 5-Phospho-alpha-D-ribose1-diphosphate | 0.316752498 | -1.658572099 | down | 0.035179757 |
|  | 6-Phospho-D-gluconate | 0.598466988 | -0.740656426 | down | 0.013544049 |
|  | Acetoacetyl-CoA | 0.367750051 | -1.443202552 | down | 0.003399493 |
|  | Acetylaminobutanal | 0.304196455 | -1.716924755 | down | 0.005529223 |
|  | Acetylornithine | 1.774272487 | 0.827227591 | up | 0.018814316 |
|  | Aconitate | 4.52541913 | 2.178051416 | up | 0.002023974 |
|  | Adenine | 0.041046153 | -4.606609182 | down | 0.001282923 |
|  | Adenosine | 0.053621694 | -4.221039394 | down | 0.01721998 |
|  | ADP | 0.048134836 | -4.376774802 | down | 0.008125134 |
|  | Alanine | 0.122809063 | -3.025511059 | down | 0.008672571 |
|  | Aminocyclopropane carboxylate | 0.509670018 | -0.972364607 | down | 0.001984063 |
|  | AMP | 0.019129984 | -5.708020494 | down | 0.004854243 |
|  | Anthranilate | 0.363979937 | -1.458069164 | down | 0.016997871 |
|  | Arginine | 0.221492256 | -2.174671834 | down | 0.005382979 |
|  | Asparagine | 0.351239023 | -1.509474955 | down | 0.030285252 |
|  | Aspartate | 0.124164404 | -3.009676461 | down | 0.003841761 |
|  | beta-D-Glucose6-phosphate | 0.020167789 | -5.631803296 | down | 0.002594069 |
|  | CDP | 0.154848418 | -2.691071447 | down | 0.002145833 |
|  | cGMP | 0.047577479 | -4.393577366 | down | 0.02367068 |
|  | cis-2-Methylaconitate | 0.070165155 | -3.833101453 | down | 0.010540779 |
|  | Citrulline | 0.339504534 | -1.558497252 | down | 0.010050474 |
|  | coenzyme A | 0.177099993 | -2.497363943 | down | 0.014892697 |
|  | Cytidine | 0.152367693 | -2.714371058 | down | 0.04879156 |
|  | Cytosine | 0.315420966 | -1.664649535 | down | 0.006067783 |
|  | d-Ala-d-Ala | 0.12228835 | -3.031641123 | down | 0.001639266 |
|  | dAMP | 0.057900716 | -4.110275 | down | 0.007787798 |
|  | Deoxyadenosine | 0.05615496 | -4.15444274 | down | 0.04006557 |
|  | Deoxyguanosine | 0.053621694 | -4.221039394 | down | 0.01721998 |
|  | Deoxyinosine | 0.04745823 | -4.397197886 | down | 0.013732303 |
|  | Deoxyribose phosphate | 3.148765102 | 1.654786136 | up | 0.006540596 |
|  | D-Erythrose 4-phosphate | 0.029997043 | -5.059035909 | down | 0.002532256 |
|  | dGDP | 0.048134836 | -4.376774802 | down | 0.008125134 |
|  | dGMP | 0.019129984 | -5.708020494 | down | 0.004854243 |
|  | dTMP | 0.062083603 | -4.009643913 | down | 0.001395739 |
|  | dUDP | 0.031506455 | -4.988208776 | down | 0.008383714 |
|  | D-Xylulose 5-phosphate | 0.029990418 | -5.059354581 | down | 0.049068447 |
|  | F6P | 0.017525639 | -5.834389156 | down | 0.026315519 |
|  | FAD | 0.064752763 | -3.948914438 | down | 0.002709322 |
|  | FGAR | 0.118286412 | -3.079643735 | down | 0.008372305 |
|  | FMN | 0.020215779 | -5.628374397 | down | 0.001018496 |
|  | G1P | 0.017525639 | -5.834389156 | down | 0.026315519 |
|  | G6P | 0.020167789 | -5.631803296 | down | 0.002594069 |
|  | Galactitol | 0.074486914 | -3.746869194 | down | 0.004074229 |
|  | Galactonate | 0.078035062 | -3.679733699 | down | 0.019631199 |
|  | Galactono-1,4-lactone | 0.556562255 | -0.845385025 | down | 0.008198837 |
|  | gamma-L-Glutamyl-L-cysteine | 0.121004279 | -3.046870031 | down | 0.025438296 |
|  | GlcNAc-P | 0.031489289 | -4.988995024 | down | 0.00550328 |
|  | Glutamine | 0.07073919 | -3.821346491 | down | 0.00739149 |
|  | Glycerate | 0.644916912 | -0.632814793 | down | 0.027011794 |
|  | Glycine | 0.180507973 | -2.469865535 | down | 0.000134973 |
|  | GMP | 0.039222118 | -4.672188738 | down | 0.002522551 |
|  | Guanine | 0.104726817 | -3.255297179 | down | 0.001677929 |
|  | Histidine | 0.405260349 | -1.303079066 | down | 0.022826263 |
|  | Homoserine | 0.135265153 | -2.886137877 | down | 0.000296773 |
|  | Hypoxanthine | 0.025678528 | -5.283293712 | down | 0.004276674 |
|  | IMP | 0.00303031 | -8.36631895 | down | 0.002335638 |
|  | Inosine | 0.017693691 | -5.82062116 | down | 0.003329538 |
|  | Isoleucine | 0.358286734 | -1.480813467 | down | 0.039098179 |
|  | Lactaldehyde | 0.129790457 | -2.945743783 | down | 0.001404755 |
|  | Leucine | 0.358286734 | -1.480813467 | down | 0.039098179 |
|  | Lysine | 0.29566968 | -1.757941787 | down | 0.00540855 |
|  | Mannosylglycerate | 0.017693691 | -5.82062116 | down | 0.003329538 |
|  | meso-Diaminopimelate | 0.332414202 | -1.588946073 | down | 0.008814557 |
|  | Methionine | 0.02834251 | -5.140888662 | down | 0.001220016 |
|  | Methylmaleate | 4.485662415 | 2.165321049 | up | 0.002175599 |
|  | NAD+ | 0.280511303 | -1.833869191 | down | 0.022915178 |
|  | NADP+ | 0.234475834 | -2.092488855 | down | 0.04485213 |
|  | Nicotinate | 0.093600278 | -3.417343369 | down | 0.011294291 |
|  | Ornithine | 0.317005187 | -1.657421646 | down | 0.01128048 |
|  | OXG | 0.066829134 | -3.903379012 | down | 0.011396319 |
|  | pantothenate | 0.141389995 | -2.822248056 | down | 0.000191042 |
|  | Phe | 0.302807492 | -1.723527196 | down | 0.014663317 |
|  | Proline | 0.179359388 | -2.479074838 | down | 0.01375206 |
|  | PYR | 0.042475448 | -4.557227035 | down | 0.040086268 |
|  | Riboflavin | 0.045383505 | -4.461688157 | down | 0.003177018 |
|  | Ribose phosphate | 0.029990418 | -5.059354581 | down | 0.049068447 |
|  | Saccharopine | 0.186525311 | -2.422556681 | down | 0.041738083 |
|  | S-Acetyldihydrolipoamide-E | 0.170992859 | -2.547992014 | down | 0.016170604 |
|  | Sedoheptulose 7-phosphate | 0.038277207 | -4.707370617 | down | 0.002594869 |
|  | SUC | 0.136386435 | -2.874227935 | down | 0.001229296 |
|  | Threonine | 0.135265153 | -2.886137877 | down | 0.000296773 |
|  | Thymidine | 0.051711257 | -4.273377812 | down | 0.001514099 |
|  | Thymine | 0.104528185 | -3.258036094 | down | 8.45289E-05 |
|  | Trehalose | 0.002691286 | -8.537488707 | down | 0.003374538 |
|  | Trp | 0.363682468 | -1.459248714 | down | 0.022643694 |
|  | Tyr | 0.139986065 | -2.836644875 | down | 0.009962383 |
|  | Ubiquinone | 0.04534586 | -4.462885359 | down | 0.012720595 |
|  | UDP | 0.03168135 | -4.980222373 | down | 0.010693328 |
|  | UDP-D-galactose | 0.01012247 | -6.626294808 | down | 0.00948433 |
|  | UDP-GlcNAc | 0.009574197 | -6.706632734 | down | 0.006154846 |
|  | UDP-glucose | 0.01012247 | -6.626294808 | down | 0.00948433 |
|  | UMP | 0.021343365 | -5.550068537 | down | 0.007848403 |
|  | Uracil | 0.062658905 | -3.996336622 | down | 0.001424572 |
|  | Urate | 0.227435952 | -2.136467771 | down | 0.000801047 |
|  | Uridine | 0.118586048 | -3.075993809 | down | 0.003533225 |
|  | Xanthine | 0.284638347 | -1.812798055 | down | 0.004373016 |
|  | Xanthosine | 0.12726471 | -2.974095677 | down | 0.000508114 |
| BC vs BDQ | (R)-2,3-Dihydroxy-3-methylbutanoate | 0.35 | -1.51 | down | 0.000746025 |
|  | 2-Aminoacrylate | 0.39 | -1.36 | down | 0.010687605 |
|  | 2-Deoxy-D-ribose 1-phosphate | 2.88 | 1.53 | down | 0.00523636 |
|  | 3-Methyl-2-oxobutanoic acid | 0.47 | -1.09 | down | 0.019347592 |
|  | 5-Methylthioadenosine | 0.16 | -2.64 | down | 0.004777704 |
|  | Acetylaminobutanal | 0.45 | -1.15 | down | 0.047850884 |
|  | Acetylornithine | 0.37 | -1.43 | down | 0.001597474 |
|  | Adenine | 0.43 | -1.22 | down | 0.049294662 |
|  | Adenosine | 0.39 | -1.36 | down | 0.037939954 |
|  | ADP | 0.12 | -3.06 | down | 0.047814097 |
|  | Alanine | 0.24 | -2.06 | down | 5.90153E-05 |
|  | AMP | 0.35 | -1.51 | down | 0.000159011 |
|  | Arginine | 0.52 | -0.94 | down | 0.028702913 |
|  | Aspartate | 0.31 | -1.69 | down | 0.001969022 |
|  | beta-D-Glucose6-phosphate | 0.43 | -1.22 | down | 0.002293146 |
|  | cAMP | 0.61 | -0.71 | down | 0.025940087 |
|  | cGMP | 0.42 | -1.25 | down | 0.003970354 |
|  | cis-2-Methylaconitate | 0.44 | -1.18 | down | 0.005440866 |
|  | Citrulline | 0.38 | -1.4 | down | 0.015142215 |
|  | coenzyme A | 0.26 | -1.94 | down | 0.02472576 |
|  | Cytidine | 0.4 | -1.32 | down | 0.000192975 |
|  | d-Ala-d-Ala | 0.4 | -1.32 | down | 0.003221822 |
|  | Deoxyadenosine | 0.47 | -1.09 | down | 0.012291048 |
|  | Deoxyguanosine | 0.39 | -1.36 | down | 0.037939954 |
|  | Deoxyinosine | 0.36 | -1.47 | down | 0.017707984 |
|  | Deoxyribose | 0.35 | -1.51 | down | 0.000746025 |
|  | Deoxyribose phosphate | 2.88 | 1.53 | up | 0.00523636 |
|  | D-Erythrose 4-phosphate | 0.64 | -0.64 | down | 0.035285501 |
|  | dGDP | 0.09 | -3.47 | down | 0.003835295 |
|  | dGMP | 0.35 | -1.51 | down | 0.014003902 |
|  | dUDP | 0.37 | -1.43 | down | 0.00014785 |
|  | F6P | 0.39 | -1.36 | down | 0.000260688 |
|  | FAD | 0.66 | -0.6 | down | 0.022123958 |
|  | FGAR | 0.6 | -0.74 | down | 0.026518372 |
|  | FMN | 0.29 | -1.79 | down | 0.004166546 |
|  | G1P | 0.39 | -1.36 | down | 0.000260688 |
|  | G6P | 0.43 | -1.22 | down | 6.52162E-05 |
|  | Galactonate | 0.55 | -0.86 | down | 0.012571328 |
|  | Galactono-1,4-lactone | 0.36 | -1.47 | down | 0.000327683 |
|  | Glutamine | 0.24 | -2.06 | down | 0.007954996 |
|  | GMP | 0.32 | -1.64 | down | 0.018755892 |
|  | Guanosine | 0.23 | -2.12 | down | 0.003648695 |
|  | Homoserine | 0.62 | -0.69 | down | 0.018832606 |
|  | Inosine | 0.51 | -0.97 | down | 0.023728052 |
|  | Lysine | 0.44 | -1.18 | down | 0.038450715 |
|  | Mannosylglycerate | 0.51 | -0.97 | down | 0.023728052 |
|  | meso-Diaminopimelate | 0.51 | -0.97 | down | 0.030186991 |
|  | Methionine | 0.57 | -0.81 | down | 0.03003155 |
|  | Methylcitrate | 0.42 | -1.25 | down | 0.003671953 |
|  | NAD+ | 0.36 | -1.47 | down | 2.20835E-05 |
|  | Nicotinamide | 0.34 | -1.56 | down | 0.014983581 |
|  | Nicotinate | 0.35 | -1.51 | down | 0.002284583 |
|  | Ornithine | 0.38 | -1.4 | down | 0.000348386 |
|  | OXG | 0.55 | -0.86 | down | 0.019195564 |
|  | Propanoyl-CoA | 1.61 | 0.69 | down | 0.043810362 |
|  | PYR | 0.51 | -0.97 | down | 0.001859319 |
|  | R5P | 0.61 | -0.71 | down | 0.02626883 |
|  | Riboflavin | 0.26 | -1.94 | down | 0.007700693 |
|  | Saccharopine | 0.3 | -1.74 | down | 0.011659783 |
|  | Sedoheptulose 7-phosphate | 0.46 | -1.12 | down | 0.002694365 |
|  | Threonine | 0.62 | -0.69 | down | 0.018832606 |
|  | Trehalose | 0.27 | -1.89 | down | 0.004011119 |
|  | Ubiquinone | 0.32 | -1.64 | down | 0.014969838 |
|  | UDP-D-galactose | 0.22 | -2.18 | down | 0.009788319 |
|  | UDP-GlcNAc | 0.27 | -1.89 | down | 0.000724331 |
|  | UDP-glucose | 0.22 | -2.18 | down | 0.009788319 |
|  | UMP | 0.53 | -0.92 | down | 0.036056503 |
|  | Uracil | 0.29 | -1.79 | down | 0.031259093 |
|  | Urate | 0.55 | -0.86 | down | 0.024976218 |
|  | Uridine | 28.73 | 4.84 | up | 0.011566869 |
|  | Xanthine | 0.58 | -0.79 | down | 0.02551313 |
|  | Xanthosine | 0.55 | -0.86 | down | 0.027463301 |
| BC vs CUR | (R)-2,3-Dihydroxy-3-methylbutanoate | 0.34 | -1.56 | down | 0.000209197 |
|  | 2-Aminoacrylate | 0.47 | -1.64 | down | 0.00487875 |
|  | 2-Oxobutanoate | 1.55 | 0.63 | down | 0.006264537 |
|  | 2-Phospho-D-glycerate | 0.23 | -2.12 | down | 0.040041614 |
|  | 3-Oxopropanoate | 0.14 | -2.84 | down | 0.000587343 |
|  | 5-Methylthioadenosine | 0.02 | -5.64 | down | 0.0167081 |
|  | 6-Phospho-D-gluconate | 0.31 | -1.69 | down | 0.009245046 |
|  | Acetylaminobutanal | 0.48 | -1.79 | down | 0.000917747 |
|  | Acetylornithine | 2.16 | 1.11 | down | 0.011931722 |
|  | Aconitate | 2.46 | 1.3 | up | 0.0036474 |
|  | Adenine | 0.08 | -4.06 | down | 0.007463547 |
|  | Adenosine | 0.11 | -3.47 | down | 0.006783646 |
|  | ADP | 0.06 | -4.06 | down | 0.001929643 |
|  | Alanine | 0.36 | -2.4 | down | 7.2071E-05 |
|  | AMP | 0.04 | -4.64 | down | 0.000766651 |
|  | Arginine | 0.31 | -1.69 | down | 4.30752E-05 |
|  | Aspartate | 0.28 | -2.32 | down | 0.000210307 |
|  | beta-D-Glucose6-phosphate | 0.03 | -5.64 | down | 0.001514698 |
|  | CDP | 0.12 | -3.06 | down | 0.010210918 |
|  | cGMP | 0.05 | -4.32 | down | 0.002618587 |
|  | cis-2-Methylaconitate | 0.15 | -2.74 | down | 2.83245E-05 |
|  | Citrulline | 0.35 | -1.6 | down | 0.013787018 |
|  | coenzyme A | 0.15 | -2.94 | down | 0.042381107 |
|  | Cytidine | 0.29 | -3.47 | down | 0.021918002 |
|  | Cytosine | 0.5 | -1 | down | 0.003506187 |
|  | d-Ala-d-Ala | 0.16 | -3.64 | down | 0.002786691 |
|  | dAMP | 0.11 | -3.18 | down | 0.004351991 |
|  | Deoxyadenosine | 0.09 | -3.64 | down | 0.01079123 |
|  | Deoxyguanosine | 0.11 | -3.47 | down | 0.006783646 |
|  | Deoxyinosine | 0.02 | -5.64 | down | 0.0109624 |
|  | Deoxyribose | 0.34 | -1.56 | down | 0.000209197 |
|  | D-Erythrose 4-phosphate | 0.04 | -4.64 | down | 8.32376E-07 |
|  | dGDP | 0.06 | -4.06 | down | 0.001929643 |
|  | dGMP | 0.04 | -4.64 | down | 0.003350866 |
|  | dTMP | 0.13 | -2.94 | down | 0.002096419 |
|  | dUDP | 0.05 | -4.32 | down | 0.002518822 |
|  | D-Xylulose 5-phosphate | 0.05 | -4.32 | down | 0.003039187 |
|  | F6P | 0.02 | -5.64 | down | 0.001776611 |
|  | FAD | 0.13 | -3.47 | down | 0.013413786 |
|  | FGAR | 0.21 | -2.25 | down | 0.041688682 |
|  | FMN | 0.02 | -5.64 | down | 0.000816202 |
|  | G1P | 0.02 | -5.64 | down | 0.001776611 |
|  | G6P | 0.03 | -5.06 | down | 9.10677E-05 |
|  | Galactitol | 0.13 | -2.94 | down | 0.000592665 |
|  | Galactonate | 0.12 | -3.06 | down | 0.001106712 |
|  | gamma-L-Glutamyl-L-cysteine | 0.19 | -2.4 | down | 0.0038405 |
|  | GAR | 2.7 | 1.43 | up | 0.049642968 |
|  | GlcNAc-P | 0.05 | -4.32 | down | 0.002563799 |
|  | Glutamine | 0.06 | -4.64 | down | 0.003633348 |
|  | Glycine | 0.27 | -2.25 | down | 0.001592896 |
|  | GMP | 0.06 | -4.06 | down | 0.005552922 |
|  | Guanine | 0.39 | -2.32 | down | 0.009990908 |
|  | Guanosine | 0.09 | -3.47 | down | 0.025536312 |
|  | Histidine | 0.46 | -1.18 | down | 0.047938375 |
|  | Homoserine | 0.23 | -2.74 | down | 0.000877223 |
|  | IMP | 0.01 | -6.64 | down | 0.00078283 |
|  | Inosine | 0.16 | -5.06 | down | 0.026051178 |
|  | Isoleucine | 0.62 | -0.69 | down | 0.01656902 |
|  | Lactaldehyde | 0.18 | -2.47 | down | 0.000773712 |
|  | Leucine | 0.62 | -0.69 | down | 0.018829081 |
|  | Lysine | 0.47 | -1.18 | down | 0.00769409 |
|  | Mannosylglycerate | 0.16 | -5.06 | down | 0.026051178 |
|  | meso-Diaminopimelate | 0.25 | -3.64 | down | 0.047776983 |
|  | Methionine | 0.07 | -4.06 | down | 0.000204305 |
|  | Methylcitrate | 3.53 | 1.82 | down | 0.048049952 |
|  | Methylmaleate | 2.43 | 1.28 | down | 0.004512128 |
|  | NAD+ | 0.05 | -4.32 | down | 0.000783965 |
|  | NADP+ | 0.31 | -1.69 | down | 0.031680544 |
|  | Nicotinate | 0.14 | -3.06 | down | 0.02416298 |
|  | Ornithine | 0.33 | -1.64 | down | 0.004486493 |
|  | OXG | 0.17 | -2.56 | down | 0.001086306 |
|  | pantetheine | 0.32 | -1.64 | down | 0.003912285 |
|  | pantothenate | 0.34 | -1.56 | down | 0.025276475 |
|  | Phe | 0.46 | -1.12 | down | 0.001000534 |
|  | Proline | 0.37 | -1.43 | down | 0.000177571 |
|  | PYR | 0.09 | -3.47 | down | 0.002627017 |
|  | R5P | 0.05 | -4.32 | down | 0.003056057 |
|  | Riboflavin | 0.08 | -3.84 | down | 0.008364019 |
|  | Ribose phosphate | 0.05 | -4.32 | down | 0.003039187 |
|  | Saccharopine | 0.27 | -1.89 | down | 0.000589478 |
|  | Sedoheptulose 7-phosphate | 0.06 | -4.06 | down | 0.000554141 |
|  | SUC | 0.19 | -2.4 | down | 0.000410475 |
|  | Succinate semialdehyde | 1.55 | 0.63 | down | 0.006264537 |
|  | Threonine | 0.23 | -2.74 | down | 0.000877223 |
|  | Thymidine | 0.06 | -4.06 | down | 0.000847804 |
|  | Thymine | 0.07 | -3.84 | down | 0.001148714 |
|  | Trehalose | 0.004 | -12.2020474 | down | 0.000212235 |
|  | Trp | 0.49 | -1.03 | down | 0.000239845 |
|  | Tyr | 0.2 | -2.32 | down | 0.002002714 |
|  | Ubiquinone | 0.07 | -3.84 | down | 0.001367625 |
|  | UDP | 0.28 | -3.84 | down | 0.016841369 |
|  | UDP-D-galactose | 0.02 | -5.64 | down | 0.003696049 |
|  | UDP-glucose | 0.02 | -5.64 | down | 0.003696049 |
|  | UDP-GlcNAc | 0.17 | -5.64 | down | 0.046270352 |
|  | UMP | 0.1 | -4.64 | down | 0.003953963 |
|  | Uracil | 0.1 | -3.32 | down | 2.63245E-05 |
|  | Urate | 0.25 | -2 | down | 0.005074099 |
|  | Uridine | 0.08 | -3.64 | down | 0.000307124 |
|  | Xanthosine | 0.21 | -2.25 | down | 0.001047494 |
